# Poster Session II - A201 COMPARISON OF INVASIVE *HELICOBACTER PYLORI* DETECTION METHODS IN PATIENTS UNDERGOING GASTROSCOPY IN A CANADIAN TERTIARY CARE CENTER.

**DOI:** 10.1093/jcag/gwaf042.201

**Published:** 2026-02-13

**Authors:** J M Duerksen, B Ramjiawan, J K Stone

**Affiliations:** Medicine, University of Manitoba Max Rady College of Medicine, Winnipeg, MB, Canada; St Boniface Hospital Research, Winnipeg, MB, Canada; Medicine, University of Manitoba Max Rady College of Medicine, Winnipeg, MB, Canada

## Abstract

**Background:**

Helicobacter pylori (HP) is a prevalent gastrointestinal bacterium that predisposes individuals to a variety of gastrointestinal disorders. Various invasive testing methods are available including culture, rapid urease testing (RUT) and histopathology, however no gold standard exists and there is heterogeneity in the accuracy of these across various studies.

**Aims:**

To compare the accuracy of three invasive diagnostic techniques (histopathology, RUT and culture) for *HP* and determine predictors of positive results for each. Our secondary aim is to characterize the prevalence of *HP* infection in Manitoba

**Methods:**

Retrospective single center study of adult patients undergoing gastroscopy and biopsy for *HP* testing. For each patient, biopsies were taken for histopathological, RUT and culture. Patient demographics including age, sex, and medication use (PPI, H2RA, antibiotics and anticoagulants/NSAIDs) were collected. Procedure related data collected included indication for gastroscopy, endoscopic findings and biopsy results. The presence of HP and active or inactive gastritis was reported. Regression analysis was performed to determine predictors of positive findings on histopathology, RUT, culture and HP prevalence.

**Results:**

Of the 142 cases analyzed there were 30 positive *H. pylori* results. 29 (96.6%) were positive by histopathology, 17(56.6%) were positive by culture, and only 1(3.3%) was positive by RUT. One case that was not positive by histopathology was positive by culture. All positive cases were accompanied by either active or inactive gastritis. After multivariate regression analysis, only age >50 years was predictive of histopathology (OR 1.17, 95% CI 1.01,1.34; p = 0.036) and prevalence (OR 1.17, 95% CI 1.02,1.36; p = 0.028). The prevalence of *HP* in our study was 21.1%. Using histopathology as the gold standard, we report a culture sensitivity of 58.6% and specificity of 92.3%.

**Conclusions:**

Histopathology shows the highest accuracy for diagnosing HP in our single center study, despite previous literature suggesting RUT as a highly sensitive and specific test for *H. pylori* detection. RUT had the lowest accuracy of the three tests we assessed which may be due to site-specific factors and should therefore be used with caution. Age was the only predictive variable of HP prevalence and histopathologic positivity.

**Funding Agencies:**

St. Boniface Research Center, Winnipeg, MB.

